# Calendar

**DOI:** 10.1155/S1463924686000093

**Published:** 1986


### Calendar

### MARCH 1986

### Pittsburgh Conference and Exposition on Analytical Chemistry and Applied Spectroscopy

To be held from 10–14 March in Atlantic City, New Jersey, USA.

*Contact George L. Vassilaros, Registration Chairman, 12 Federal Drive, Suite 322, Pittsburgh, Pennsylvania 15235, USA.*

### APRIL

### Gas Liquid Chromatography

Course to be held from 7–11 April in Loughborough, UK.

*Contact Dr R. M. Smith, Department of Chemistry, University of Technology, Loughborough, Leicestershire LE11 3TU, UK.*

### Fourth World Filtration Congress

To be held from 22 to 25 April 1986 in Ostend, Belgium.

*Contact Secretariat of World Filtration Congress, IV, KVIV-Technologisch Instituut, Jan van Rijswijcklaan 58, B 2018 Antwerpen, Belgium.*

### Separations for Biotechnology and Biochemistry

Course to be held from 21–25 April in Loughborough, UK.

*Contact Dr R. M. Smith (above).*

### MAY

### Tenth International Symposium on Column Liquid Chromatography

To be held at the Sheraton Palace Hotel, San Francisco, California, USA from 18 to 23 May.

*Contact Ms Shirley Schlessinger, Symposium Manager of HPLC 86, 400 E. Randolph Drive, Chicago, Illinois 60601, USA.*

### JUNE

### Analytica 86

To be held from 3 to 6 June at München, FR Germany.

*Contact Münchener Messe-und Austellungsgesellschaft mbH, Messegelände, Postfach 12 10 09, D8000 München 12, FR Germany.*

### Annual Congress of the Canadian Society of Laboratory Technologists

To be held from 15 to 20 June in St John's, Newfoundland, Canada.

*Contact CSLT, PO Box 830, Hamilton, Ontario, Canada L8N 3N8.*

### Third International Congress on Paediatric Laboratory Medicine

To be held from 29 June to 3 July in Bristol, UK.

*Contact Mrs Anne Green, Department of Clinical Chemistry, The Children's Hospital, Ladywood, Middleway, Birmingham B16 8ET, UK.*

### JULY

### Statistics for Analytical Chemistry

Course to be held from 7–10 July in Loughborough, UK.

*Contact Dr R. M. Smith (above).*

### FIA

Course to be held from 7–11 July in Loughborough, UK.

*Contact Dr R. M. Smith (above).*

### National Meeting of the AACC

To be held from 13 to 18 July in Chicago, USA.

*Contact AACC, 1725 K Street, N.W., Suite 1010, Washington, D.C. 20006.*

### SAC 86/3rd BNASS: an International Conference on Analytical Chemistry

To be held from 20–26 July 1986 at the University of Bristol, UK.

*Contact The Secretary, Analytical Division, Royal Society of Chemistry, Burlington House, London W1V 0BN.*

### AUGUST

### Sixth International Congress of Pesticide Chemistry

To be held from 10–15 August in Ottawa, Canada.

*Contact H. V. Morley, Chairman: Organizing Committee. The Chemical Institute of Canada, 151 Slater Street, Suite 906, Ottawa, Ontario, Canada K1P 5H3.*

### SEPTEMBER

### 3rd International Conference on Measurement in Clinical Medicine

To be held from 9–11 September in Edinburgh, UK.

*Contact the Institute of Measurement and Control, 87 Gower Street, London WC1E 6AA.*

### HPLC

Course to be held from 15–19 September in Loughborough, UK.

*Contact Dr R. M. Smith (above).*

### 3rd African, Mediterranean and Near East Congress of Clinical Chemistry

To be held from 28 September to 2 October in Sevilla, Spain.

*Contact Apartado de Correos 6209, 41080 Sevilla, Spain.*

### OCTOBER

### The Silver Jubilee Eastern Analytical Symposium

To be held from 6–10 October in New York, USA.

*Contact Dr S. David Klein, EAS Publicity, 642 Cranbury Cross Road, North Brunswick, New Jersey 08902, USA.*

